# Supplementary material for: Predicting visual function by interpreting a neuronal wiring diagram
Source: Nature. 2024 Oct 2;634(8032):113–23. doi: 10.1038/s41586-024-07953-5 (PMC11446822; doi:10.1038/s41586-024-07953-5)
Supplement: Supplementary file 6 — CRF and ERF predictions for individual TmY4 and TmY9 cells. Analogous to Supplementary Data 3, but for TmY target types. Shown are the top four monosynaptic pathways, the strongest pathway passing through each of the top ten intermediary types (ranking from Extended Data Fig. 7), and the trisynaptic pathway Tm1–TmY–Dm3–TmY (see the section entitled Prediction of spatial normalization). [file 41586_2024_7953_MOESM6_ESM.zip › DataS4/TmY9qΓèÑ/720575940631613259.pdf]

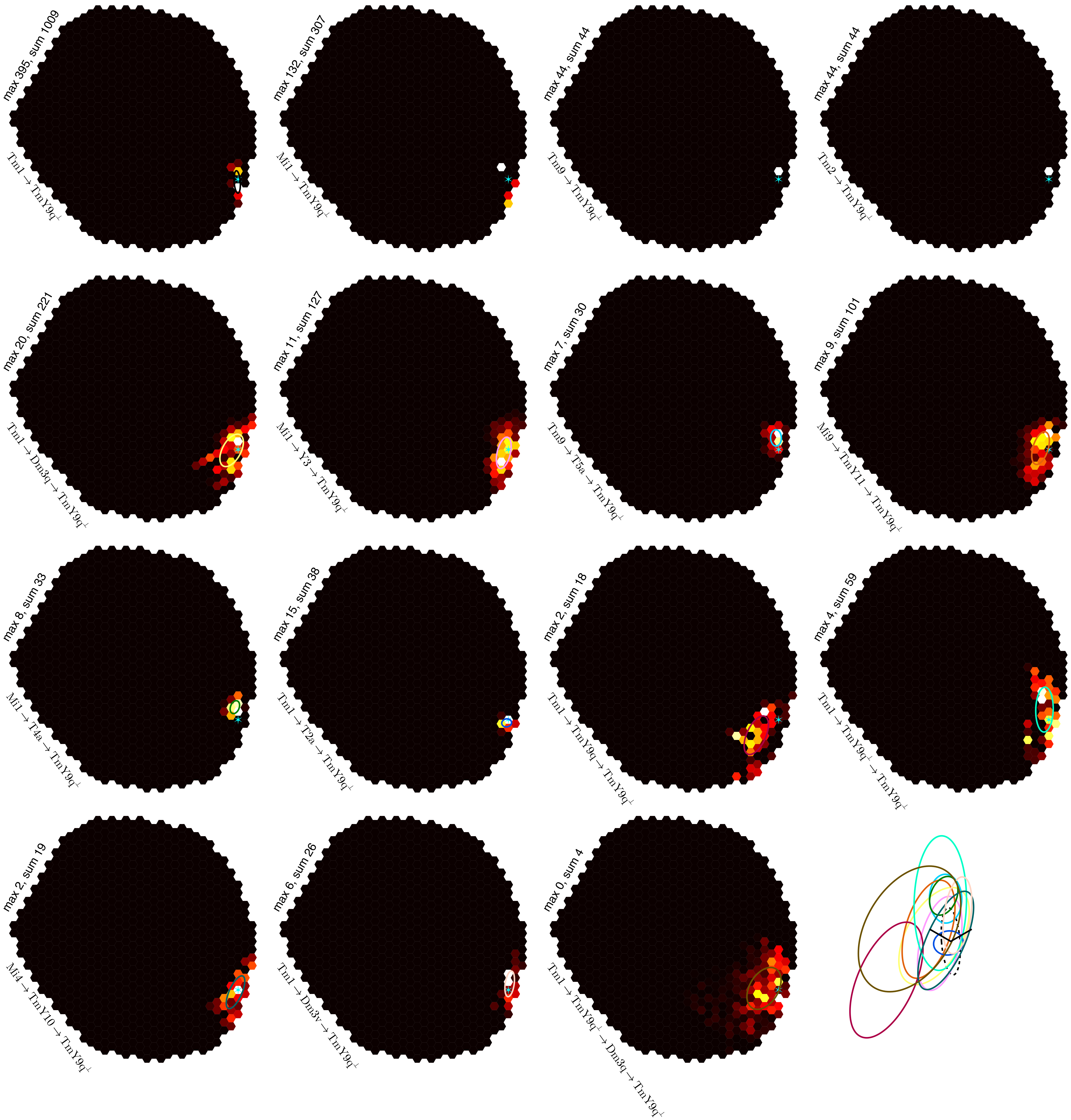
max 395, sum 1009
Tm1 → TmY9q⊥
max 132, sum 307
Mi1 → TmY9q⊥
max 44, sum 44
Tm9 → TmY9q⊥
max 44, sum 44
Tm2 → TmY9q⊥
max 20, sum 221
Tm1 → Dm3q → TmY9q⊥
max 11, sum 127
Mi1 → Y3 → TmY9q⊥
max 7, sum 30
Tm9 → T5a → TmY9q⊥
max 9, sum 101
Mi9 → TmY11 → TmY9q⊥
max 8, sum 33
Mi1 → T4a → TmY9q⊥
max 15, sum 38
Tm1 → T2a → TmY9q⊥
max 2, sum 18
Tm1 → TmY9q → TmY9q⊥
max 4, sum 59
Tm1 → TmY9q⊥ → TmY9q⊥
max 2, sum 19
Mi4 → TmY10 → TmY9q⊥
max 6, sum 26
Tm1 → Dm3v → TmY9q⊥
max 0, sum 4
Tm1 → TmY9q⊥ → Dm3q → TmY9q⊥
